# Sociodemographic, medical, health behavior, and psychosocial factors associated with COVID-19 diagnoses in the New Jersey cancer survivor cohort

**DOI:** 10.1007/s10552-025-01997-2

**Published:** 2025-04-25

**Authors:** Sharon Manne, Adana A. M. Llanos, Hari S. Iyer, Lisa E. Paddock, Katie Devine, Shawna V. Hudson, Denalee O’Malley, Elisa V. Bandera, Sara Frederick, Jacintha Peram, Justin Solleder, Shengguo Li, Hao Liu, Andrew M. Evens

**Affiliations:** 1 Rutgers Cancer Institute of New Jersey, 120 Albany Street, Tower 2 Floor 8, New Brunswick, NJ 08901 USA; 2 Department of Epidemiology, Mailman School of Public Health, Columbia University Irving Medical Center, 722 West 168th Street, New York, NY 10032 USA; 3 Herbert Irving Comprehensive Cancer Center, Columbia University Irving Medical Center, 722 West 168th Street, New York, NY 10032 USA; 4 Cancer Epidemiology and Health Outcomes, Rutgers Cancer Institute of New Jersey, 120 Albany Street, Tower 2 Floor 8, New Brunswick, NJ 08901 USA; 5 Cancer Surveillance Research Program, Cancer Epidemiology Services, NJ Department of Health, New Jersey State Cancer Registry, Rutgers Cancer Institute of New Jersey, PO Box 369, Trenton, NJ 08625-0369 USA; 6 Rutgers Cancer Institute of New Jersey, 195 Little Albany Street, New Brunswick, NJ 08901 USA; 7 Department of Family Medicine and Community Health, Rutgers Robert Wood Johnson Medical School, 303 George Street, Rm 309, New Brunswick, NJ 08901 USA; 8 Division of Biometrics, Rutgers Cancer Institute of New Jersey, 195 Little Albany Street, New Brunswick, NJ 08901 USA; 9 Department of Biostatistics and Epidemiology, Rutgers School of Public Health, 120 Albany St, New Brunswick, NJ 08901 USA

**Keywords:** Cancer survivors, COVID, Social vulnerability, Social determinants

## Abstract

**Background:**

Cancer survivors are more susceptible to contracting COVID-19. However, beyond race, age, and sex, less is known about other neighborhood and psychosocial factors contribute to this increased risk.

**Objective:**

The goal of this study was to examine the associations of individual and area-level social determinants of health (SDOH) measures, medical, lifestyle, and psychosocial factors and COVID-19 infection in a statewide cohort of cancer survivors in New Jersey.

**Methods:**

Survey data from 864 cancer survivors in New Jersey were collected from 2018 to 2022, which were merged with study participant data from the state of New Jersey on COVID-19 diagnoses in 2020, 2021, and 2022. We estimated adjusted odds ratios (aOR) for associations of COVID-19 diagnosis with individual-level factors (cancer type and stage, health behaviors, and psychosocial factors) and area-level SDOH [Social Vulnerability Index, Area Deprivation Index, and Index of Concentration at the Extremes (ICE) to quantify racialized deprivation vs. privilege based on income].

**Results:**

Cancer survivors born outside the US were more than twice as likely to contract COVID-19 compared to US-born survivors (aOR 2.29, 95% CI 1.01, 4.92). Compared to Quartile 4, residence in an area in Quartile 1 of racialized income ICE (i.e., predominantly Black, low income) was associated with higher odds of COVID-19 (aOR 2.15, 95% CI 0.98, 4.87). Retired survivors had lower odds of COVID-19 (aOR 0.39, 95% CI 0.19, 0.80) compared to those who were employed. Higher social well-being was associated with higher COVID-19 (aOR 1.07, 95% CI 1.02, 1.13). Type of cancer and cancer treatments received were not associated with the risk of COVID-19.

**Conclusions:**

Immigrant status and increased racialized deprivation as measured by ICE for income were associated with COVID-19. These findings support evidence that individual *and* area-level SDOH measures contribute to increased risk of COVID-19 among cancer survivors.

**Supplementary Information:**

The online version contains supplementary material available at 10.1007/s10552-025-01997-2.

As of September 2024, more than 11 million confirmed cases of COVID-19 and more than 1.2 million COVID-19-related deaths were documented in the United States (US) [1]. Studies have shown that persons with a recent cancer diagnosis are more susceptible to contracting COVID [2–12] and are at higher risk for complications such as hospitalization and death [6, 13–18]. Among cancer survivor populations, little is known about the factors that are associated with increased risk of COVID-19 infection beyond reported for cancer type [16, 19], race and ethnicity [19], sex [19], and age [19, 20]. A greater understanding of these factors could assist in efforts to mitigate modifiable risk factors among cancer patients and survivors to reduce exposures to future COVID-19 infection.

Major factors associated with COVID-19 infections that have been documented in the general population include race and ethnicity. In the general population, elevated risks of COVID-19 infection, hospitalization, and death have been reported among racial and ethnic minorities, particularly those self-identifying as non-Hispanic Black (NHB) and Hispanic, compared to non-Hispanic White (NHW) individuals [21, 22]. However, other medical, lifestyle, and employment factors as well as social and structural drivers of health (SDOH, both at the individual-level and area-level) [23], may contribute to exposure risk. Factors identified in the general cancer literature associated with poorer cancer-related outcomes may also correlate with elevated risk of COVID-19 infection. In terms of SDOH, these include lower education, income, nativity and birthplace, neighborhood-level vulnerability factors (e.g., social vulnerability, census-level area deprivation) and cancer-related financial hardships may be important to evaluate [24–31]. Possible medical factors associated with increased risk of COVID-19 infection and severity among cancer survivors include multimorbidity, cancer \\\\type, and cancer stage. Lifestyle factors include tobacco, alcohol use, low physical activity, high body mass index (BMI), and psychosocial factors such as fear of recurrence and quality of life, which may influence engagement in COVID-19 preventative methods, may also be important to evaluate [32].

In this study, we examined the association of a broad array individual and area-level SDOH measures, medical, lifestyle, and psychosocial factors and COVID-19 infection in a statewide cohort of cancer survivors recruited into a survey study during between October 2018 and March 2022.

## Methods

The methods and measures have been described in previous work published from this study [33–35].

### Eligibility

Participants were drawn from the New Jersey Survivorship Cohort Study database, which evaluated survivorship care experiences among New Jersey cancer survivors recruited by the New Jersey State Cancer Registry (NJSCR) [33–35]. Participant eligibility criteria were: (a) 18–85 years of age; (b) a current NJ resident; (c) diagnosed in 2015, 2016, or 2018 with a primary case of genitourinary (i.e., bladder and prostate), female breast, gynecologic (i.e., cervical, endometrial, ovarian), colorectal, lung, melanoma, or thyroid cancer and; (d) able to read and speak English. The NJSCR is a population-based cancer registry and the sample represented New Jersey’s (NJ) cancer population by using proportional stratified random sampling to select cases using county, race, ethnicity, gender, and cancer type strata. Races other than white and Hispanic ethnicity were oversampled to account for typically lower participation in these populations. Counties were grouped into regions for further analysis: North NJ, Central NJ, and South NJ.

### Procedures and participation

Institutional review board approval was obtained from the Rutgers Institutional Review Board and conformed with the standards of United States Federal Policy for the Protection of Human Subjects. After potential subjects were identified through the NJSCR, they were confirmed by a Certified Tumor Registrar and a recruitment package was mailed. Individuals were called by NJSCR staff to confirm receipt and answer questions. Surveys were completed and mailed back. Prospective participants were called once or twice a week, with a maximum of eight calls. Patients who could be reached were labeled passive refusers. Patients who were contacted and refused participate were labeled active refusers. Recruitment packets were re-sent up to three times during the recruitment period. Completed questionnaires were considered a consent per Rutgers Institutional Review Board written informed consent waiver approval. Participants received a $25 gift card.

Between August 2018 and January 2022, 3,348 individuals met the initial eligibility and were contacted. Five hundred thirty-eight were not eligible, 1,830 refused, 116 were unable to be contacted (incorrect address/phone), and 864 returned the survey (31.9% response rate). Comparisons of the 864 acceptors and the 1,830 refusers based on available data (sex, race, ethnicity, age, cancer type, cancer stage) indicated that Hispanic survivors were more likely to decline participation (75.5%) than non-Hispanic survivors (67.5%) (Chi-square = 4.4, *p* < 0.05) In addition, there were significant differences in cancer type (Chi-square = 20.4, *p* < 0.001), with breast cancer survivors having the lowest refusal rate (61.8%) and thyroid cancer survivors having the highest refusal rate (74.8%).

Cancer survivors in the New Jersey Survivorship Cohort Study were linked to New Jersey Department of Health COVID-19 cases collected through the Communicable Disease Reporting and Surveillance System (CDRSS). CDRSS is an electronic, web-enabled system where public health partners statewide can instantly report and track communicable disease incidence using data from electronic laboratory reporting transmissions or manual entries from clinical laboratories and acute care hospitals. Public Law 116–136, § 18,115 (a), the Coronavirus Aid, Relief, and Economic Security (CARES) Act, has required “every laboratory that performed or analyzed a test intended to detect SARS-CoV-2 or to diagnose a possible case of COVID-19” to report the results, positive and negative, from each such test to public health authorities, including at-home specimen collection. Match*Pro linkage software (v1.65, 2.3), a free software available from the National Cancer Institute (https://seer.cancer.gov/tools/matchpro/), was used to perform probabilistic record linkages between individuals diagnosed with cancer and COVID based on the Fellegi and Sunter model. First name, last name, birth date, street address, phone, gender, and date of death were used as matching variables. Accuracy was extensively used to validate match status of a subset and to adjudicate uncertain pairs. Over 1.5 million PCR-confirmed COVID-19 cases diagnosed among NJ residents in 2020–2021 were used in the linkage. Vaccination data were included from the NJ Immunization Information System (NJIIS). NJSCR staff conducted quality control procedures and provided a limited dataset to the study team.

### Social and structural drivers of health (SDOH) measures

Available data for self-identified sex (male or female), race [White, Black or African American, Asian or Asian American, or other (including American Indian or Alaska Native, Native Hawaiian or other Pacific Islander, and multiracial)], and Hispanic ethnicity (yes or no) were included. If self-reported values were missing, then the values reported to the NJSCR were used. Age at cancer diagnosis was calculated as the difference between the patient’s birthdate and date of cancer diagnosis. Age was categorized as < 65, 65–75, < 75 years. Nativity was assessed by asking if the survivor was born in the US [yes or no]. Marital status was coded as [married (including separated couples), unmarried (divorced or widowed), or single/never married].

#### Socioeconomic status (SES)

SES was assessed by education [high school graduate or less, some college/post high school, college graduate, or post college (graduate degree, professional degree, or other certifications/credits following a college degree)], employment status (employed/self-employed, unemployed/homemaker/student/disabled, or retired), annual household income (< $50,000, $50,000–$89,999, and ≥ $90,000), and health insurance status [uninsured, private, public (Medicaid or Medicare), or insured–not otherwise specified].

#### Neighborhood socioeconomic disadvantage

The Area Deprivation Index (ADI) is based on a measure originally created by the Health Resources and Services Administration (HRSA) adapted and validated to the Census Block Group neighborhood-level [36, 37]. The ADI allows for rankings of neighborhoods by socioeconomic disadvantage in a region of interest [38]. It includes four domains (income, education, employment, and housing quality) which were captured at Block Group level in 2015 to measure pre-COVID lockdown-related influences of neighborhood disadvantage. To calculate an ADI, census data block group data are ranked in percentiles from 1 to 100 (1 = lowest disadvantage within the nation to 100 = highest level of disadvantage.

#### Index of concentration at the extremes

The index of concentration at the extremes (ICE) captures concentration of income and racial privilege vs disadvantaged and has been used as a proxy for segregation and adverse impacts of structural racism [39, 40]. The three most-commonly used ICE measures capture racial segregation (concentration of White vs. Black residents); income (concentration of residents in the highest vs lowest quintile of household income); and jointly across race and income (concentration of White residents in the highest quintile of income vs. Black residents in the lowest quintile of income). ICE measures range from − 1 to 1, with values closer to 1 indicating higher privilege. ICE measured were calculated using data at the census tract-level from the 2006–2010 American Community Survey. The ICE is associated with higher incidence and poorer survival for many cancers [40–42].

#### Social vulnerability index

The census tract-level social vulnerability index (SVI) was developed by the agency for toxic substances and disease registry (ATSDR) Geospatial Research, Analysis and Services Program with the intent of identifying community-level susceptibilities to disasters (severe weather, floods, disease outbreaks, chemical contamination) [43]. It relies on fifteen measures derived from the US Census capturing social factors (socioeconomic status, household composition and disability, minoritized status and language, and housing type and transportation access). The Index is calculated numerically and as a percentile, with higher values indicating greater vulnerability. The SVI is of particular relevance given the well documented adverse impact of the COVID-19 pandemic in socially vulnerable populations [44, 45].

#### Cancer-related financial hardship

This measure had five items: Borrowing money/go into debt, cannot not afford medications, declared bankruptcy, unable to cover cost of care visit, and worry about paying bills (*yes/no*) [46, 47]. Because this variable was highly skewed, for this analysis, the scale was dichotomized to 0 and 1.

### Medical variables

#### Comorbidities

A checklist of 23 health conditions derived from the Health Information National Trends Survey (HINTS) [48] was included in the questionnaire to assess patients’ history of cardiovascular and metabolic (0, 1, or ≥ 2), pulmonary (0 or ≥ 1), and other (0 or ≥ 1) comorbidities. Cardiovascular and metabolic comorbidities included a history of diabetes, hypertension, hypercholesterolemia, heart disease/angina/heart attack, congestive heart failure, myocardial infarction, kidney disease, and/or liver disease; pulmonary comorbidities included a history of emphysema/chronic obstructive pulmonary disease (COPD), and/or asthma; and other comorbidities included a history of depression, anxiety disorder, schizophrenia, bipolar disorder, post-traumatic stress disorder (PTSD), peripheral vascular disease. Cerebrovascular disease, dementia, connective tissue disease, leukemia, malignant lymphoma, hematological or solid tumor, and/or acquired immunodeficiency syndrome (AIDS). Total, cardiovascular/metabolic and pulmonary comorbidities were included in the analyses.

#### Cancer history

Participants self-reported their primary cancer diagnosis [female breast, colorectal, genitourinary (urinary bladder or prostate), gynecologic (vulvar, endocervical, cervical, endometrial, myometrium, corpus uteri, and ovarian), lung, malignant skin (melanoma), or thyroid]. Cancer stage was determined based on the year of diagnosis and staging mechanism; patients were classified as unstaged, early stage (in situ and localized cases), or late stage (regional and distant cases). Time (in years) since cancer diagnosis was calculated as the difference between the date of cancer diag vbnnosis and date of survey completion. Receipt of cancer treatment (surgery, chemotherapy, or radiation therapy) was recorded (yes, no, or do not recall).

### Health behavior variables

#### Current alcohol use

The Follow-up Care Use Health Outcomes Survey (FOCUS) questionnaire [49] item assessing alcohol consumption [49] was used [“Have you had any beer, wine, wine coolers, mixed drinks, liquor, or other alcoholic beverages during the past month?” (*yes, no, don’t know, prefer not to answer*)].

#### Tobacco use

Current smoking status was assessed by an item from the FOCUS survey [49] [How often do you smoke cigarettes now?” (every day, some days, not at all)]. Use was categorized as yes/no.

#### Physical activity

Physical activity was measured using the Godin Leisure Time Exercise Questionnaire. Four items measure assess the number of sessions per week when participant engages in 30 min or more of mild, moderate, and strenuous activity. Scores below 14 were classified as insufficiently active/sedentary, scores from 14 to 23 were classified as moderately active, and scores of 24 or higher were classified as active.

#### Body mass index (BMI)

BMI was calculated from self-reported height and weight as weight in kg divided by the square of height. Participants were characterized as underweight (< 18.5), normal weight (18.5–24.9), overweight (25–29.9), or obese (> 30). Underweight was combined with normal weight for analyses, due to the small percentage of participants who were underweight.

### Psychosocial variables

#### Fear of recurrence

Participants completed the Concerns about Recurrence Scale [50], a 4-item scale assessing worries about the possibility of cancer recurrence (1 = *not at all*; 6 = *extremely)*. A total average score was computed, with higher scores indicated greater fear. *Α* = 0.94.

#### Health-related quality of life (HRQoL)

The functional assessment of cancer therapy-general (FACT-G) (Version 4) [51] is a widely used patient-reported outcome instrument measuring HRQoL in cancer patients. It has four domains: Physical well-being (PWB, 7 items), social/family well-being (SWB, 7 items), emotional well-being (EWB, 7 items), and functional well-being (FWB, 7 items). Likert ratings range from *not at all* (0) to *very much* (4). Averages were computed for scale scores. The total scale and subscales were included in the analyses. Reliability for total HRQoL *α* = 0.92, and subscale reliabilities were: PWB *α* = 0.87, SWB *α* = 0.85, EWB *α* = 0.71, and FWB *α* = 0.92. Higher scores indicate better QOL on all scales.

### Data analytic approach

COVID-19 diagnosis (yes/no) during the years 2020 and 2021 as reported in NJSCR-CDRSS linked data was the dependent variable in analyses. Descriptive statistics were used to summarize the baseline characteristics for the full sample, participants who contracted COVID, and participants who did not contract COVID. The difference between COVID and non-COVID participants was analyzed using the Wilcoxon rank-sum test for continuous variables, Pearson’s Chi-squared test for categorical variables, and Fisher’s exact test for binary variables. Logistic regression models were used to examine the association of the baseline characteristics with COVID diagnosis. Given the large number of possible predictors, we adopted a two-step analytic approach. First, we conducted a univariate logistic regression. Second, based on the results from the univariate analyses, we selected variables that attained *p* < 0.05 statistical significance to include in a multivariable logistic regression.

For all area-level measures, we performed a series of analytic procedures to assess the importance of individual neighborhood measures, as well as the summary scores (ADI, ICE, SVI). We used correlation matrices to evaluate associations between component Census socioeconomic measures. For ADI, ICE, and SVI, we parameterized exposure using quartiles. Next, we fit regression models for COVID-19 diagnosis, adjusting for age, race, Hispanic ethnicity, nativity, employment status, and social well-being using approaches described above. Only racialized income ICE was retained in the final model due to high correlation between all neighborhood socioeconomic measures and because it exhibited the highest correlation with COVID diagnosis in variable selection procedures described above.

## Results

### Characteristics of the sample (Table 1)

**Table 1:** Descriptive statistics of New Jersey cancer survivors, *n* = 864

Characteristic	*n*	Overall, *n* = 864^a^	COVID, *n* = 97^a^	No COVID, = 767^a^	*p*-Value^b^
Sex	864				0.5
Male		349(40.4%)	36(37.1%)	313(40.8%)	
Female		515(59.6%)	61(62.9%)	454(59.2%)	
Race	837				0.015
White		662(79.1%)	67(73.6%)	595(79.8%)	
Black/African American		107(12.8%)	14(15.4%)	93(12.5%)	
Asian/Asian American		52(6.2%)	4(4.4%)	48(6.4%)	
Other^c^		16(1.9%)	6(6.6%)	10(1.3%)	
(Missing)		27	6	21	
Hispanic ethnicity	848				0.004
Yes		52(6.1%)	13(13.8%)	39(5.2%)	
No		787(92.8%)	79(84.0%)	708(93.9%)	
Prefer not to answer		9(1.1%)	2(2.1%)	7(0.9%)	
(Missing)		16	3	13	
Age	852				0.2
< 65		411(48.2%)	55(57.3%)	356(47.1%)	
65–75		343(40.3%)	32(33.3%)	311(41.1%)	
> 75		98(11.5%)	9(9.4%)	89(11.8%)	
(Missing)		12	1	11	
BMI	817				
Under and normal weight (BMI < 25)		245(30.0%)	30(32.6%)	215(29.7%)	
Overweight (BMI 25–30)		293(35.9%)	22(23.9%)	271(37.4%)	
Obese (BMI > 30)		279(34.1%)	40(43.5%)	239(33.0%)	
(Missing)		47	5	42	0.030
US-born	859				0.014
Yes		737(85.8%)	74(76.3%)	663(87.0%)	
No		118(13.7%)	22(22.7%)	96(12.6%)	
Prefer not to answer		4(0.5%)	1(1.0%)	3(0.4%)	
(Missing)		5	0	5	
Marital status	858				0.7
Married^d^		578(67.4%)	68(70.8%)	510(66.9%)	
Unmarried		163(19.0%)	18(18.8%)	145(19.0%)	
Never married		109(12.7%)	9(9.4%)	100(13.1%)	
Don’t know/prefer not to answer		8(0.9%)	1(1.0%)	7(0.9%)	
(Missing)		6	1	5	
Education	858				0.5
High school graduate or less		194(22.6%)	18(18.8%)	176(23.1%)	
Some college/post high school^e^		199(23.2%)	21(21.9%)	178(23.4%)	
College graduate		261(30.4%)	30(31.3%)	231(30.3%)	
Post college^f^		180(21.0%)	22(22.9%)	158(20.7%)	
Other-unspecified/don’t know/prefer not to answer		24(2.8%)	5(5.2%)	19(2.5%)	
(Missing)		6	1	5	
Employment status	847				0.017
Employed/self-employed		354(41.8%)	50(53.2%)	304(40.4%)	
Unemployed/homemaker/student/disabled		114(13.5%)	12(12.8%)	102(13.5%)	
Retired		354(41.8%)	27(28.7%)	327(43.4%)	
Other-unspecified/don’t know/prefer not to answer		25(3.0%)	5(5.3%)	20(2.7%)	
(Missing)		17	3	14	
Household income	847				0.3
< $50,000		205(24.2%)	28(29.2%)	177(23.6%)	
$50,000–$89,999		191(22.6%)	25(26.0%)	166(22.1%)	
≥ $90,000		266(31.4%)	28(29.2%)	238(31.7%)	
Don’t know		185(21.8%)	15(15.6%)	170(22.6%)	
(Missing)		17	1	16	
Health insurance status at cancer diagnosis	864				0.4
Uninsured		12(1.4%)	2(2.1%)	10(1.3%)	
Private		409(47.3%)	51(52.6%)	358(46.7%)	
Public^g^		355(41.1%)	33(34.0%)	322(42.0%)	
Insured, not otherwise specified		55(6.4%)	8(8.2%)	47(6.1%)	
Don’t know		33(3.8%)	3(3.1%)	30(3.9%)	
NJ region	864				> 0.9
Central NJ		293(33.9%)	33(34.0%)	260(33.9%)	
North NJ		285(33.0%)	32(33.0%)	253(33.0%)	
South NJ		286(33.1%)	32(33.0%)	254(33.1%)	
National level area deprivation index score^h^	858				0.4
Q1 (low disadvantage)		214(24.9%)	20(20.6%)	194(25.5%)	
Q2 (low to moderate disadvantage)		220(25.6%)	27(27.8%)	193(25.4%)	
Q3 (moderate to high disadvantage)		217(25.3%)	30(30.9%)	187(24.6%)	
Q4 (high disadvantage)		207(24.1%)	20(20.6%)	187(24.6%)	
(Missing)		6	0	6	
State level area deprivation index score^i^	778				0.6
Q1 (low disadvantage)		175(22.5%)	20(22.0%)	155(22.6%)	
Q2 (low to moderate disadvantage)		188(24.2%)	22(24.2%)	166(24.2%)	
Q3 (moderate to high disadvantage)		236(30.3%)	32(35.2%)	204(29.7%)	
Q4 (high disadvantage)		179(23.0%)	17(18.7%)	162(23.6%)	
Income ICE^j^	860				0.2
Q1 (low income)		214(24.9%)	30(30.9%)	184(24.1%)	
Q2 (low to moderate income)		215(25.0%)	25(25.8%)	190(24.9%)	
Q3 (moderate to high income)		215(25.0%)	25(25.8%)	190(24.9%)	
Q4 (high income)		216(25.1%)	17(17.5%)	199(26.1%)	
(Missing)		4	0	4	
Race ICE^k^	861				0.005
Q1 (low % white)		215(25.0%)	33(34.4%)	182(23.8%)	
Q2 (low to moderate % white)		215(25.0%)	23(24.0%)	192(25.1%)	
Q3 (moderate to high % white)		215(25.0%)	29(30.2%)	186(24.3%)	
Q4 (high % white)		216(25.1%)	11(11.5%)	205(26.8%)	
(Missing)		3	1	2	
Racialized income ICE^l^	861				0.042
Q1 (low % high income white)		215(25.0%)	31(32.0%)	184(24.1%)	
Q2 (low to moderate % high income white)		215(25.0%)	30(30.9%)	185(24.2%)	
Q3 (moderate to high % high income white)		215(25.0%)	21(21.6%)	194(25.4%)	
Q4 (high % of high income white)		216(25.1%)	15(15.5%)	201(26.3%)	
(Missing)		3	0	3	
Social vulnerability theme summary^m^	861				0.090
Q1 (low vulnerable)		216(25.1%)	18(18.6%)	198(25.9%)	
Q2 (low to moderate vulnerable)		214(24.9%)	26(26.8%)	188(24.6%)	
Q3 (moderate to high income)		216(25.1%)	20(20.6%)	196(25.7%)	
Q4 (high vulnerable)		215(25.0%)	33(34.0%)	182(23.8%)	
(Missing)		3	0	3	
Social vulnerability ranking^n^	861				0.090
Q1 (low vulnerable)		216(25.1%)	18(18.6%)	198(25.9%)	
Q2 (low to moderate vulnerable)		214(24.9%)	26(26.8%)	188(24.6%)	
Q3 (moderate to high income)		216(25.1%)	20(20.6%)	196(25.7%)	
Q4 (high vulnerable)		215(25.0%)	33(34.0%)	182(23.8%)	
(Missing)		3	0	3	
Cancer-related financial hardship^o^	861				0.4
No		460(53.4%)	47(49.0%)	413(54.0%)	
Yes		401(46.6%)	49(51.0%)	352(46.0%)	
(Missing)		3	1	2	
Cardiometabolic comorbidities^p^	864				> 0.9
0		273(31.6%)	31(32.0%)	242(31.6%)	
1		151(17.5%)	18(18.6%)	133(17.3%)	
≥ 2		440(50.9%)	48(49.5%)	392(51.1%)	
Pulmonary comorbidities^q^	864				0.3
0		700(81.0%)	75(77.3%)	625(81.5%)	
≥ 1		164(19.0%)	22(22.7%)	142(18.5%)	
Total comorbidities^r^	864				0.5
0		157(18.2%)	15(15.5%)	142(18.5%)	
≥ 1		707(81.8%)	82(84.5%)	625(81.5%)	
Primary cancer diagnosis	864				0.6
Bladder		81(9.4%)	10(10.3%)	71(9.3%)	
Breast		216(25.0%)	25(25.8%)	191(24.9%)	
Colorectal		99(11.5%)	12(12.4%)	87(11.3%)	
Gynecologic cancer		88(10.2%)	6(6.2%)	82(10.7%)	
Lung		86(10.0%)	10(10.3%)	76(9.9%)	
Melanoma		78(9.0%)	9(9.3%)	69(9.0%)	
Prostate		154(17.8%)	14(14.4%)	140(18.3%)	
Thyroid		62(7.2%)	11(11.3%)	51(6.6%)	
Cancer stage at diagnosis^s^	864				> 0.9
Early stage		707(81.8%)	79(81.4%)	628(81.9%)	
Late stage		133(15.4%)	16(16.5%)	117(15.3%)	
Unstaged		24(2.8%)	2(2.1%)	22(2.9%)	
Time from diagnosis to survey (years)	863	3.15 (0.62)	3.16 (0.70)	3.15 (0.61)	0.6
(Missing)		1	0	1	
Surgery	843				> 0.9
Yes		714(84.7%)	83(85.6%)	631(84.6%)	
No		128(15.2%)	14(14.4%)	114(15.3%)	
Do not recall		1(0.1%)	0(0.0%)	1(0.1%)	
(Missing)		21	0	21	
Chemotherapy	783				0.030
Yes		251(32.1%)	30(34.9%)	221(31.7%)	
No		529(67.6%)	54(62.8%)	475(68.1%)	
Do not recall		3(0.4%)	2(2.3%)	1(0.1%)	
(Missing)		81	11	70	
Radiotherapy	809				0.8
Yes		340(42.0%)	36(40.4%)	304(42.2%)	
No		468(57.8%)	53(59.6%)	415(57.6%)	
Do not recall		1(0.1%)	0(0.0%)	1(0.1%)	
(Missing)		55	8	47	
Last saw doctor for cancer follow-up	855				0.7
Less than 4 weeks		192(22.5%)	23(24.0%)	169(22.3%)	
1 to 3 months		262(30.6%)	24(25.0%)	238(31.4%)	
4 to 6 months		224(26.2%)	28(29.2%)	196(25.8%)	
7 to 12 months		123(14.4%)	13(13.5%)	110(14.5%)	
More than 2 year		54(6.3%)	8(8.3%)	46(6.1%)	
(Missing)		9	1	8	
Alcohol, past month	836				0.3
Yes		532(63.6%)	53(55.8%)	479(64.6%)	
No		298(35.6%)	42(44.2%)	256(34.5%)	
Don’t know		3(0.4%)	0(0.0%)	3(0.4%)	
Prefer not to answer		3(0.4%)	0(0.0%)	3(0.4%)	
(Missing)		28	2	26	
Physical activity	765				0.8
Insufficiently active		249(32.5%)	25(29.1%)	224(33.0%)	
Moderately active		141(18.4%)	17(19.8%)	124(18.3%)	
Active		375(49.0%)	44(51.2%)	331(48.7%)	
(Missing)		99	11	88	
Ever smoke	853	417(48.9%)	47(49.0%)	370(48.9%)	> 0.9
(Missing)		11	1	10	
Global fear	844	2.73 (1.45)	2.94 (1.48)	2.71 (1.45)	0.13
(Missing)		20	3	17	
Physical well-being	850	24.75(4.80)	25.13(4.21)	24.70(4.87)	0.7
(Missing)		14	1	13	
Social well-being	848	21.14(6.14)	22.37(5.69)	20.99(6.18)	0.016
(Missing)		16	2	14	
Emotional well-being	844	24.22(3.94)	24.63(3.54)	24.16(3.99)	0.3
(Missing)		20	2	18	
Functional well-being	848	21.39(6.62)	22.52(5.61)	21.24(6.72)	0.11
(Missing)		16	2	14	
HRQoL	835	13.08(2.41)	13.53(2.13)	13.02(2.44)	0.060
(Missing)		29	4	25	

^a^*n*(%); Mean (SD)

^b^Pearson’s Chi-squared test; Wilcoxon rank-sum test; Fisher’s exact test

^c^Including American Indian or Alaska Native, Native Hawaiian/Other Pacific Islander, Other. Please specify

^d^Including both married and separated

^e^Including post high school training other than college (vocational or technical)

^f^Graduate degree

^g^Also including TRICARE, Military, Veterans Affairs and Indian/Public Health

^h^1 = indicates the lowest level of disadvantage within the nation; 100 = indicates the greatest level of disadvantage within the nation, Q1: (1,15], Q2: (15,26], Q3: (26,41], Q4: (41,98]

^i^1 = indicates the lowest level of disadvantage within the state; 10 = indicates the greatest level of disadvantage within the state, Q1: (1,3], Q2: (3,5], Q3: (5,8], Q4: (8,10]

^j^(# residents in top 20th % of income—# residents in bottom 20th % of income)/total residents, Q1: (− 0.703,0.363], Q2: (0.363,0.532], Q3: (0.532,0.672], Q4: (0.672,0.948]

^k^(# White residents—# Black residents)/total residents, Q1: (− 0.941,0.651], Q2: (0.651,0.82], Q3: (0.82,0.916], Q4: (0.916,1]

^l^(# White residents in top 20th % of income—# Black residents in bottom 20th % of income)/total residents’ Q1: (− 0.589,0.383], Q2: (0.383,0.529], Q3: (0.529,0.638], Q4: (0.638,0.881]

^m^A combination of 4 different themes (Housing, Minority status/language, Household Composition, Socioeconomic), Q1: (2.07,4.9], Q2: (4.9,6.2], Q3: (6.2,7.82], Q4: (7.82,12.1]

^n^The overall percentile ranking for Social vulnerability theme summary, Q1: (0,0.202], Q2: (0.202,0.404], Q3: (0.404,0.625], Q4: (0.625,0.996]

^o^0 = No, >  = 1 = Yes

^p^Including diabetes, hypertension, hypercholesterolemia, heart disease/angina/heart attack, congestive heart failure, myocardial infarction, kidney disease, and/or liver disease

^q^Including emphysema/chronic obstructive pulmonary disease (COPD), and/or asthma

^r^Including both cardiovascular/metabolic and pulmonary comorbidities

^s^Stage 0–2 = Early stage; 3–4 = Late stage

Ninety-seven participants with cancer (11.2%) contracted COVID-19 during the study period. Descriptive statistics for the full sample are in Table 1. The majority of sample was White, non-Hispanic, US-born, and about half had completed a college level education, and carried health insurance. About 60% of the sample was female, and 67% were married. A quarter of the sample reported an annual income of $50,000 or lower, and 42% were employed. About 53% reported at least one cancer-related financial difficulty, and there was a relatively equal distribution of participants across the range of economically advantaged/disadvantaged areas of New Jersey. There were no statistically significant differences in neighborhood ADI or SVI between cancer survivors with vs without COVID-19 diagnoses. Cancer survivors resided in neighborhoods with high race ICE (Q4: 34.4% vs. 23.8%), and low racialized income ICE (32.0% vs. 24.1%).

### Univariate regression analyses

Table 2 reports the results of unadjusted OR and 95% CI using the bivariate logistic regression model. The results are also shown graphically in a forest plot in Supplementary Fig. 1. Compared to White individuals, survivors of races other than Black and Asian Americans were more likely to be diagnosed with COVID-19 [OR = 5.33, 95% CI (1.77, 14.8)]. Compared to Hispanic individuals, survivors with non-Hispanic ethnicity were less likely to be diagnosed with COVID-19 [OR = 0.33, 95% CI (0.18, 0.68)]. Compared to US-born participants, those born outside of the US were also more likely to be diagnosed [OR = 2.05, 95% CI (1.20, 3.41)]. Compared to employed survivors, retired survivors were less likely to get COVID-19 [OR = 0.50, 95% CI (0.30, 0.82)]. Survivors reporting higher social well-being were more likely to get COVID-19 [OR = 1.04, 95% CI (1.00, 1.09)].

**Table 2:** Results of the univariate logistic regressions predicting COVID infection

Variable	OR	95% CI	*p*-Value
Sex			
Male	–	–	
Female	1.17	0.76, 1.82	0.5
Race			
White	–	–	
Black/African American	1.34	0.70, 2.41	0.4
Asian/Asian American	0.74	0.22, 1.89	0.6
Other	5.33	1.77, 14.8	0.002
Hispanic ethnicity			
Yes	–	–	
No	0.33	0.18, 0.68	0.001
Age			
< 65	–	–	
65–75	0.67	0.42, 1.05	0.084
> 75	0.65	0.29, 1.31	0.3
BMI			
Under and normal (BMI < 25)	–	–	
Overweight (BMI 25–30)	0.58	0.32, 1.03	0.067
Obese (BMI > 30)	1.20	0.72, 2.01	0.5
US-born			
Yes	–	–	
No	2.05	1.20, 3.41	0.007
Marital status			
Married	–	–	
Unmarried	0.93	0.52, 1.58	0.8
Never married	0.68	0.31, 1.33	0.3
Don’t know/prefer not to answer	1.07	0.06, 6.15	> 0.9
Education			
High school graduate or less	–	–	
Some college/post high school	1.15	0.59, 2.26	0.7
College graduate	1.27	0.69, 2.39	0.4
Post college	1.36	0.71, 2.66	0.4
Other-unspecified/don’t know/prefer not to answer	2.57	0.78, 7.33	0.092
Employment status			
Employed/self-employed	–	–	
Unemployed/homemaker/student/disabled	0.72	0.35, 1.35	0.3
Retired	0.50	0.30, 0.82	0.006
Other-unspecified/don’t know/prefer not to answer	1.52	0.49, 3.95	0.4
Household income			
< $50,000	–	–	
$50,000–$89,999	0.95	0.53, 1.70	0.9
≥ $90,000	0.74	0.42, 1.30	0.3
Don’t know	0.56	0.28, 1.07	0.084
Health insurance status at diagnosis			
Uninsured	–	–	
Private	0.71	0.18, 4.72	0.7
Public	0.51	0.13, 3.42	0.4
Insured, not otherwise specified	0.85	0.18, 6.20	0.9
Don’t know	0.50	0.07, 4.20	0.5
NJ Region			
Central NJ	–	–	
North NJ	1.00	0.59, 1.67	> 0.9
South NJ	0.99	0.59, 1.67	> 0.9
National level area deprivation index score			
Q4 (high disadvantage)	–	–	
Q3 (moderate to high disadvantage)	1.50	0.83, 2.77	0.2
Q2 (low to moderate disadvantage)	1.31	0.71, 2.44	0.4
Q1 (low disadvantage)	0.96	0.50, 1.86	> 0.9
State level area deprivation index score			
Q4 (high disadvantage)	–	–	
Q3 (moderate to high disadvantage)	1.49	0.81, 2.84	0.2
Q2 (low to moderate disadvantage)	1.26	0.65, 2.50	0.5
Q1 (low disadvantage)	1.23	0.62, 2.46	0.6
Income ICE^a^			
Q4 (high income)	–	–	
Q3 (moderate to high income)	1.54	0.81, 2.99	0.2
Q2 (low to moderate income)	1.54	0.81, 2.99	0.2
Q1 (low income)	1.91	1.03, 3.64	0.044
Race ICE^a^			
Q4 (high % white vs. black)	–	–	
Q3 (moderate to high % white vs. black)	2.91	1.45, 6.23	0.004
Q2 (low to moderate % white vs. black)	2.23	1.08, 4.88	0.035
Q1 (low % white vs. black)	3.38	1.71, 7.18	< 0.001
Racialized income ICE^a^			
Q4 (high % of high income white vs. low income black)	–	–	
Q3 (moderate to high % high income white vs. low income black)	1.45	0.73, 2.94	0.3
Q2 (low to moderate % high income white vs. low income black)	2.17	1.15, 4.27	0.019
Q1 (low % high income white vs. low income black)	2.26	1.20, 4.42	0.014
Social vulnerability theme summary			
Q4 (high vulnerable)	–	–	
Q3 (moderate to high income)	0.56	0.31, 1.01	0.057
Q2 (low to moderate vulnerable)	0.76	0.44, 1.32	0.3
Q1 (low vulnerable)	0.50	0.27, 0.91	0.026
Cancer-related financial hardships			
No	–	–	
Yes	1.22	0.80, 1.87	0.4
Cardiometabolic comorbidities			
0	–	–	
1	1.06	0.56, 1.94	0.9
≥ 2	0.96	0.59, 1.56	0.9
Pulmonary comorbidities			
0	–	–	
≥ 1	1.29	0.76, 2.12	0.3
Total comorbidities			
0	–	–	
≥ 1	1.24	0.71, 2.30	0.5
Primary cancer diagnosis			
Bladder	–	–	
Breast	0.93	0.44, 2.12	0.9
Colorectal	0.98	0.40, 2.45	> 0.9
Gynecologic	0.52	0.17, 1.47	0.2
Lung	0.93	0.36, 2.41	0.9
Melanoma	0.93	0.35, 2.43	0.9
Prostate	0.71	0.30, 1.72	0.4
Thyroid	1.53	0.60, 3.94	0.4
Cancer stage at diagnosis			
Early stage	–	–	
Late stage	1.09	0.59, 1.88	0.8
Not staged	0.72	0.11, 2.52	0.7
Time from cancer diagnosis to survey (years)	1.04	0.74, 1.47	0.8
Surgery			
Yes	–	–	
No	0.93	0.49, 1.65	0.8
Chemotherapy			
Yes	–	–	
No	0.84	0.52, 1.36	0.5
Radiotherapy			
Yes	–	–	
No	1.08	0.69, 1.70	0.7
Last saw doctor for follow-up			
Less than 4 weeks ago	–	–	
1–3 months ago	0.74	0.40, 1.36	0.3
4–6 months ago	1.05	0.58, 1.90	0.9
7–12 months ago	0.87	0.41, 1.76	0.7
More than 2 year ago	1.28	0.51, 2.94	0.6
Alcohol, last month			
Yes	–	–	
No	1.48	0.96, 2.28	0.074
Physical activity			
Insufficiently active	–	–	
Moderately active	1.23	0.63, 2.35	0.5
Active	1.19	0.71, 2.03	0.5
Ever smoke			
No	–	–	
Yes	1.00	0.65, 1.54	> 0.9
Global fear	1.12	0.97, 1.29	0.13
Physical well-being	1.02	0.98, 1.07	0.4
Social well-being	1.04	1.00, 1.09	0.040
Emotional well-being	1.03	0.98, 1.10	0.3
Functional well-being	1.03	1.00, 1.07	0.076
HRQoL	1.10	1.00, 1.22	0.059

*OR* odds ratio, *CI* confidence interval, *NJ* New Jersey, *HRWQoL* health-related quality of life

^a^See Table 1 footnote

Compared to survivors residing in Quartile 4 of racialized income ICE (high concentration of high income White residents), those residing in neighborhoods with higher concentrations of low income Black residents had higher odds of COVID-19 [Quartile 2: OR = 2.17, 95% CI (1.15, 4.27); and Quartile 1: OR = 2.26, 95% CI (1.20, 4.42)]. Compared to survivors living in a neighborhood with high SVI (Quartile 4), those living in a neighborhood with low SVI were less likely to contract COVID-19 [OR = 0.50, 95% CI (0.27, 0.91)]. National and level area deprivation indexes (ADI) were not significantly associated with contracting COVID-19.

### Multivariable regression analyses

**Fig. 1:**
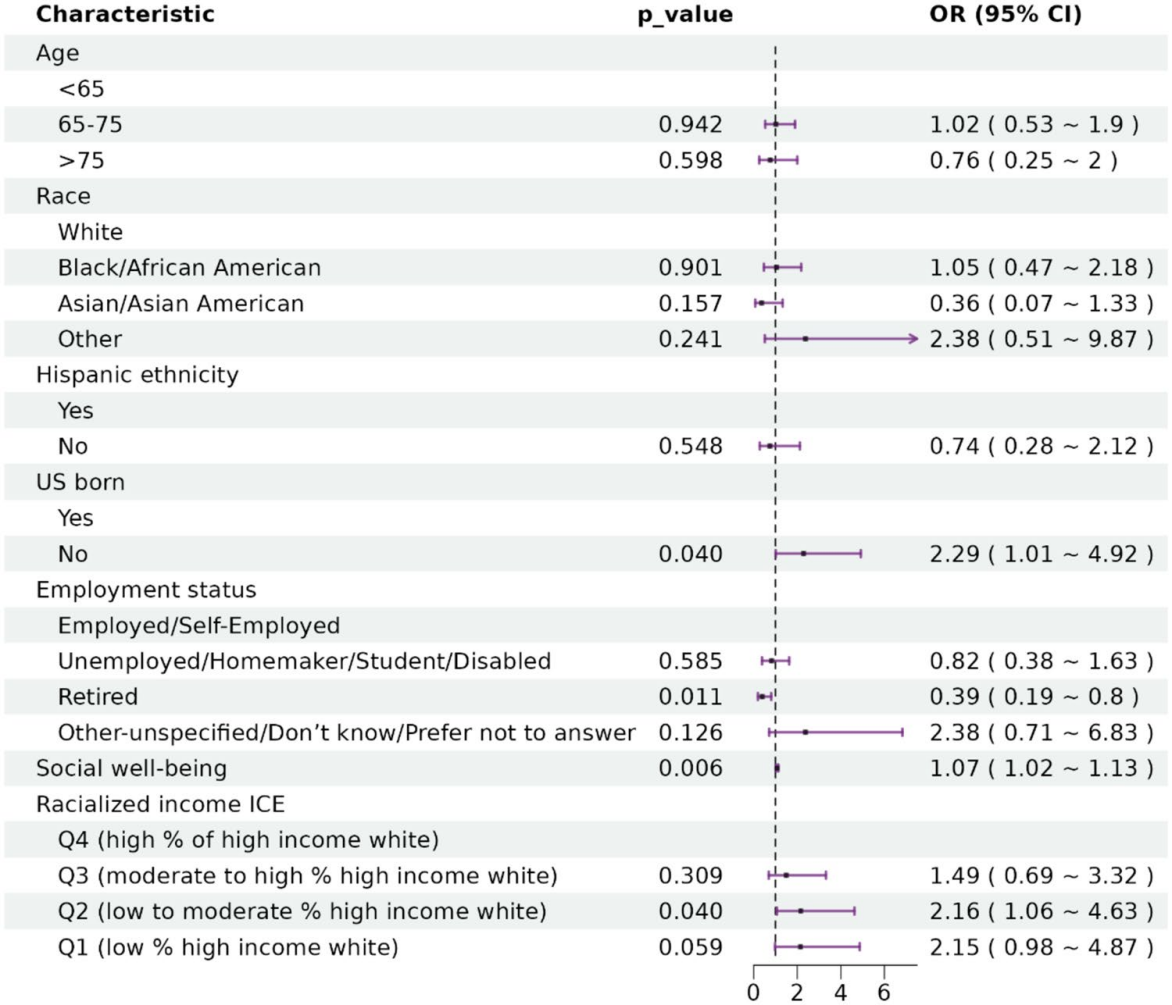
The forest plot representation of the results of the multivariable logistic regression. OR odds ratio, CI confidence interval, ICE the index of concentration at the extremes

The results of multivariable regression analysis are shown in the forest plot of the OR and 95% CI in Fig. 1 and Supplementary Table 1. Because the neighborhood measures were highly correlated within each category (ICE, SVI, ADI), we chose variables to include in the multivariable model in a stepwise fashion by taking one neighborhood measure in each domain. In the multivariable model, compared to US-born survivors, those born outside of the US had more than twofold greater odds of being diagnosed with COVID-19 (adjusted OR = 2.29, 95% CI (1.02, 4.92)]. Survivors with higher social well-being had 7% greater odds of being diagnosed with COVID-19 [adjusted OR = 1.07, 95% CI (1.02, 1.12)]. Compared to employed survivors, those who were retired were 61% less likely to contract COVID-19 [adjusted OR = 0.39, 95% CI (0.19, 0.80)]. After covariate adjustment, survivors residing in areas characterized by higher racialized income ICE had higher odds of COVID-19 diagnosis [Q1 (most disadvantaged) vs. Q4 adjusted OR = 2.15, 95% CI (0.98, 4.87), Q2 vs. Q4: adjusted OR = 2.16, 95% CI (1.06, 4.63), Q3 vs. Q4 adjusted OR = 1.49, 95% CI (0.69, 3.32)], although not all associations reached statistical significance.

## Discussion

The risk of contracting COVID-19 is higher among cancer survivors [2–18], and survivors are at elevated risk for COVID-19-related complications including death. Identifying factors associated with the risk of contracting COVID-19 using retrospective analyses can increase the understanding of who is more vulnerable and inform future responses if another outbreak and/or pandemic occurs. However, there is limited literature on this topic [16, 19, 20]. Limitations include use of non-population-based sampling approaches (e.g., hospital registries) [20, 52], a focus only on a specific cancers [53, 54], or only evaluated mortality [14, 55]. To address these limitations, we integrated data multiple sources. We linked population-based state cancer registry data with COVID-19 cases collected through CDRSS, self-reported sociodemographic, medical, lifestyle, and psychosocial information, and area-level SDOH measures capturing residential neighborhoods associated with racial segregation and unequal social privilege.

There were four significant findings. First, compared to US-born survivors, survivors born outside the US were more than twice as likely to contract COVID-19. Nativity has not been evaluated in the existing cancer survivor-focused studies. However, it is consistent with existing research in the general population illustrating a disparity in COVID-19 diagnoses among immigrants [56–58] Other than insurance status (all participants in this study were insured), factors associated with elevated COVID-19 risk among non-US-born cancer survivors are the same as those for other disparities in COVID risks for foreign-born individuals in the general population: they are more likely to work in low-skill occupations, where social distancing or remote work are not feasible [59–62], less able to advocate for safer working conditions based on their immigrant status, and more likely to reside in more crowded living arrangements with other household members who also work in a low-skill occupations. Second, survivors residing in areas with higher racialized income ICE had higher odds of contracting COVID-19. Racialized income ICE has not been previously evaluated in the existing cancer survivor-focused studies of COVID-19. However, other studies have demonstrated associations of race and racial residential segregation with COVID-19 in the general population [63–69].

The third significant finding was that retired survivors were less likely to contract COVID-19 than employed survivors. This is somewhat surprising given the well-established association of age with COVID-19 mortality. Prior cancer-focused studies and has been measured differently in studies of COVID-19 risk in the general population (e.g., low wage jobs/essential/public contact) [62, 70]. One explanation for this finding is that there are increased COVID-19 exposures among survivors who are still in the workforce. Finally, higher social well-being was associated with an increased probability of contracting COVID-19. This variable has not been considered in prior cancer-focused or general population studies. It is possible that the measure of social well-being evaluated, which assessed closeness and support from family and friends, was a proxy measure for close contact with others, which placed them at higher exposure risk. As we did not measure the degree to which participants limited exposure to large groups of family and friends, practiced masking or social distancing, or received COVID-19 vaccines, it would be difficult to explain this finding without additional data on possible exposure and engagement in COVID risk mitigation.

It is also important to note other factors that were not associated with COVID-19 that have been reported in other work. In contrast to our findings, other investigations have reported increased COVID-19 risk among certain cancers (e.g., leukemia, non-Hodgkin’s lymphoma) [16, 19, 20]. We did not find that sex, age, income, education, employment status, BMI, and smoking status were associated with getting COVID-19. In studies focusing on the general population, male sex [71], older age [70, 72, 73] less education [74], have been associated with higher risk for getting COVID-19. Recent reviews of COVID and cancer have pointed out that individual-level income data has not supported an association with COVID-19 diagnosis [53]. Higher BMI has been associated with COVID-19 severity, but not diagnosis in the general population [75]. Finally, associations between smoking and COVI9-19 in the general population have been inconsistent [76, 77].

A key strength of our study is integration of self-reported information with data collected by population-based state cancer registry. Cancer data from the NJSCR meets or exceeds cancer registry standards for quality. COVID-19 diagnoses were documented by each state during the time period of the patient-facing survey study, which allowed for the study of survivors on their survey responses. In terms of limitations, despite attempts to recruit a diverse population that was representative of the state’s composition, our sample was not as diverse as expected: Our sample was primarily comprised of individuals who self-identified as NHW (79%), which is higher than New Jersey’s profile (51% NHW) [78]. Percentage of non-US-born survivors (13.7%) was lower than figures reported by New Jersey (23%). Although we attempted to oversample racial and ethnic subgroups, many did not return the survey. Given the disparity in COVID-19 rates, oversampling racialized minorities may have produced different results. Furthermore, only the most acculturated Hispanics, who were proficient in English to respond to the questionnaire, participated in the survey. Therefore, the impact of COVID-19 in the Hispanic cancer survivor population was probably underestimated in our study. Data on vaccination uptake and social distancing practices were not collected from the entire sample and limits the interpretation of our findings. Finally, we could not conduct analyses focusing only on the cancer survivors with COVID-19 to examine disparities in complications because reliable data on COVID complications was not available in the NJSCR-CDRSS linked database.

Despite these limitations, this relatively large cohort of New Jersey cancer survivors with completed measures of individual, neighborhood, cancer-related, health behavior, and psychosocial variables during the COVID-19 pandemic provided an opportunity to characterize factors associated with COVID-19 diagnoses among cancer survivors. This study identified survivors at increased risk for contracting COVID-19 who might benefit from enhanced information and attention about strategies to reduce the risk of COVID-19 exposure. Future research on COVID-19 risk factors among cancer survivors could benefit from including a broader approach to assessing social vulnerability factors as well as other potential psychosocial risk factors.

## Supplementary Information

Below is the link to the electronic supplementary material.

Supplementary file1 (DOCX 61 KB)

Supplementary file2 (DOCX 1209 KB)

## Data Availability

The data sets generated and/or analyzed during the current study are not publicly availability because it contains New Jersey COVID and Cancer Registry data. However, they may be availability for the corresponding author upon reasonable request.
