# Supplementary material for: Sociodemographic, medical, health behavior, and psychosocial factors associated with COVID-19 diagnoses in the New Jersey cancer survivor cohort
Source: Cancer Causes Control. 2025 Apr 25;36(8):853–70. doi: 10.1007/s10552-025-01997-2 (PMC12289746; doi:10.1007/s10552-025-01997-2)
Supplement: Supplementary file 1 — Supplementary file1 (DOCX 61 KB) [file 10552_2025_1997_MOESM1_ESM.docx]

**Supplemental Materials: Survey**

What is your age? years old

What is your biological sex?

❑ Male ❑ Female

❑ Don’t know ❑ Prefer not to answer

Are you Hispanic, Latino/a, or Spanish origin? Check **all** that apply.

❑ No

| Are you…. | **Yes** |
| --- | --- |
| Mexican, Mexican American, Chicano/a | ❏ |
| Puerto Rican | ❏ |
| Dominican | ❏ |
| Another Hispanic, Latino/a or Spanish origin  Other, please specify: | ❏ |

❑ Yes

❑ Don’t know

❑ Prefer not to answer

Which of the following describes your race? Check **all** that apply.

|  | **Yes** |
| --- | --- |
| White | ❏ |
| Black or African American | ❏ |
| American Indian or Alaska Native | ❏ |
| Asian or Asian American | ❏ |
| Native Hawaiian/Other Pacific Islander | ❏ |
| Other  If other, please specify | ❏ |

If Asian or Asian American, please specify (check all that apply):

|  | **Yes** |
| --- | --- |
| Asian Indian | ❏ |
| Chinese | ❏ |
| Filipino | ❏ |
| Japanese | ❏ |
| Korean | ❏ |
| Vietnamese | ❏ |
| Other Asian | ❏ |
| If Other Asian, please specify: | |

Were you born in the United States?

❑ Yes ❑ No

❑ Don’t know ❑ Prefer not to answer

If not, where were you born?

What is your current marital status?

❑ Married or living as married ❑Divorced

❑ Widowed ❑Separated

❑ Single, never been married ❑Don’t know

❑ Prefer not to answer

What is the highest grade or level of schooling you completed?

❑ Less than 8 years

❑ 8 through 11 years

❑ High school graduate or GED

❑ Post high school training other than college (vocational or technical)

❑ Some college

❑ College graduate

❑ Graduate degree

❑ Other, please specify:

❑ Don’t know

❑ Prefer not to answer

Which of the following categories best describes your **household income**?

❑ Less than $10,000 ❑ $60,000 to $69,999

❑ $10,000 to $19,999 ❑ $70,000 to $79,999

❑ $20,000 to $29,999 ❑ $80,000 to $89,999

❑ $30,000 to $39,999 ❑ $90,000 or more

❑ $40,000 to $49,999 ❑ I don’t know

❑ $50,000 to $59,999 ❑ I prefer not to answer

**Cancer related financial hardship**

Have you or has anyone in your family had to borrow money or go into debt because of your cancer, its treatment, or the lasting effects of that treatment?

❑ Yes

❑ No

Did you or your family ever file for bankruptcy because of your cancer, its treatment, or the

lasting effects of that treatment?

❑ Yes ❑ No

Have you or your family had to make any other kinds of financial sacrifices because of your

cancer, its treatment, or the lasting effects of that treatment?

❑ Yes ❑ No

Have you ever worried about having to pay large medical bills related to your cancer?

❑ Yes ❑ No

Have you ever been unable to cover your share of the cost of medical care visits for cancer, its treatments, or lasting effects of that treatment?

❑ Yes ❑ No

## Quality of Life

## How much do you agree with the following statements about how you’ve felt in the last month …

| **Physical Well-being** | **Not at all** | **A little**  **bit** | **Some- what** | **Quite a bit** | **Very much** |
| --- | --- | --- | --- | --- | --- |
| I have a lack of energy | ❑ | ❑ | ❑ | ❑ | ❑ |
| I have nausea | ❑ | ❑ | ❑ | ❑ | ❑ |
| Because of my physical condition, I have trouble meeting the needs of my family | ❑ | ❑ | ❑ | ❑ | ❑ |
| I have a pain | ❑ | ❑ | ❑ | ❑ | ❑ |
| I am bothered by side effects of treatment | ❑ | ❑ | ❑ | ❑ | ❑ |
| I feel ill | ❑ | ❑ | ❑ | ❑ | ❑ |
| I am forced to spend time in bed | ❑ | ❑ | ❑ | ❑ | ❑ |

**How much do you agree with the following statements about how you’ve felt in the last month…**

| **Social/Family Well-being** | **Not**  **at all** | **A little**  **bit** | **Some-**  **what** | **Quite**  **a bit** | **Very**  **much** |
| --- | --- | --- | --- | --- | --- |
| I feel close to my friends | ❑ | ❑ | ❑ | ❑ | ❑ |
| I get emotional support from my family | ❑ | ❑ | ❑ | ❑ | ❑ |
| I get support from my friends | ❑ | ❑ | ❑ | ❑ | ❑ |
| My family has accepted my illness | ❑ | ❑ | ❑ | ❑ | ❑ |
| I am satisfied with family communication about my illness | ❑ | ❑ | ❑ | ❑ | ❑ |
| I feel close to my partner (or the person who is my main support) | ❑ | ❑ | ❑ | ❑ | ❑ |
| *Regardless of your current level of sexual activity, please answer the following question. If you prefer not to answer it, please check this box* ❑*and go to the next section.* | ❑ | ❑ | ❑ | ❑ | ❑ |
| I am satisfied with my sex life |  |  |  |  |  |

## How much do you agree with the following statements about how you’ve felt in the last month…

| **Emotional Well-being** | **Not at all** | **A little bit** | **Some- what** | **Quite a bit** | **Very much** |
| --- | --- | --- | --- | --- | --- |
| I feel sad | ❑ | ❑ | ❑ | ❑ | ❑ |
| I am satisfied with how I am coping with my illness | ❑ | ❑ | ❑ | ❑ | ❑ |
| I am losing hope in the fight against my illness | ❑ | ❑ | ❑ | ❑ | ❑ |
| I feel nervous | ❑ | ❑ | ❑ | ❑ | ❑ |
| I worry about dying | ❑ | ❑ | ❑ | ❑ | ❑ |
| I worry that my condition will get worse | ❑ | ❑ | ❑ | ❑ | ❑ |
| I have thoughts of hurting myself or that I would be better off dead | ❑ | ❑ | ❑ | ❑ | ❑ |

**How much do you agree with the following statements about how you’ve felt in the last month…**

| **Functional Well-being** | **Not at all** | **A little bit** | **Some- what** | **Quite a bit** | **Very much** |
| --- | --- | --- | --- | --- | --- |
| I am able to work (include work at home) | ❑ | ❑ | ❑ | ❑ | ❑ |
| My work (include work at home) is fulfilling | ❑ | ❑ | ❑ | ❑ | ❑ |
| I am able to enjoy life | ❑ | ❑ | ❑ | ❑ | ❑ |
| I have accepted my illness | ❑ | ❑ | ❑ | ❑ | ❑ |
| I am sleeping well | ❑ | ❑ | ❑ | ❑ | ❑ |
| I am enjoying the things I usually do for fun | ❑ | ❑ | ❑ | ❑ | ❑ |
| I am content with the quality of my life right now | ❑ | ❑ | ❑ | ❑ | ❑ |

**Fear of Recurrence**

How much time do you spend thinking about the possibility that your cancer could recur?

| 1 | 2 | 3 | 4 | 5 | 6 |
| --- | --- | --- | --- | --- | --- |
| I do not think  about it at all |  |  |  |  | I think about it  all the time |

How much does the possibility that your cancer could recur upset you?

| 1 | 2 | 3 | 4 | 5 | 6 |
| --- | --- | --- | --- | --- | --- |
| It does not  upset me |  |  |  |  | It makes me  extremely upset |

How often do you worry about the possibility that your cancer could recur?

| 1 | 2 | 3 | 4 | 5 | 6 |
| --- | --- | --- | --- | --- | --- |
| I never worry  about it |  |  |  |  | I worry about it  all the time |

How afraid are you that your cancer may recur?

| 1 | 2 | 3 | 4 | 5 | 6 |
| --- | --- | --- | --- | --- | --- |
| I never worry  about it |  |  |  |  | I worry about it  all the time |

**Height and Weight**

What is your current height, without shoes? feet inches

❑ Don’t know ❑ Prefer not to answer

What is your current weight, without shoes? pounds

❑ Don’t know ❑ Prefer not to answer

**Physical Activity**

During a **typical week** (7-day period), on average how many **times per week** do you do the following kinds of exercise for **more than 15 minutes during your free time?** (On each line, write the appropriate number)

1. **Strenuous Exercise** (heart beats rapidly)

Times per week

e.g. running, jogging, hockey, football, soccer, squash, basketball, cross country skiing, judo, roller skating, vigorous swimming, vigorous long distance bicycling

1. **Moderate Exercise** (not exhausting)

Times per week

e.g. fast walking, baseball, tennis, easy bicycling, volleyball, badminton, easy swimming, alpine walking, popular and folk dancing

1. **Mild Exercise** (minimal effort)

Times per Week

e.g. yoga, archery, fishing from river bank, bowling, horseshoes, golf, snowmobiling, easy walking

During a **typical week** (7-day period), in your **leisure time**, how often do you engage in any regular activity **long enough to work up a sweat** (heart beats rapidly)? Check one answer.

❑ 3 or more times per week ❑ 2 times per week ❑ 0-1 times per week

**Tobacco Use**

How often do you smoke cigarettes **now**?

❑ Every day

❑ Some days

❑ Not at all

**Alcohol use**

Have you had any beer, wine, wine coolers, mixed drinks, liquor or other alcoholic

beverages **during the past month?**

❑ Yes ❑ No

❑ Don’t know ❑ Prefer not to answer

**Comorbidities**

Have you **ever** been told by a doctor or other health professional that you have any of the

following? ***If yes*,** please provide **month** and **year of diagnosis** and whether you have taken

**the medication** for the condition in the **past 6 months.**

|  | **Diagnosis** | | ***If yes*** | **Medications**  ***Within past 6 months*** | |
| --- | --- | --- | --- | --- | --- |
|  | **Yes** | **No** | ***MM/YY*** | **Yes** | **No** |
| Diabetes or Sugar Diabetes | ❏ | ❏ |  | ❏ | ❏ |
| Asthma | ❏ | ❏ |  | ❏ | ❏ |
| High blood pressure | ❏ | ❏ |  | ❏ | ❏ |
| Emphysema or Chronic Bronchitis (COPD) | ❏ | ❏ |  | ❏ | ❏ |
| Heart Disease, Angina, or Heart Attack | ❏ | ❏ |  | ❏ | ❏ |
| Depression | ❏ | ❏ |  | ❏ | ❏ |
| Anxiety Disorder | ❏ | ❏ |  | ❏ | ❏ |
| Schizophrenia | ❏ | ❏ |  | ❏ | ❏ |
| Bipolar Disorder | ❏ | ❏ |  | ❏ | ❏ |
| Post-Traumatic Stress Disorder (PTSD) | ❏ | ❏ |  | ❏ | ❏ |
| High cholesterol | ❏ | ❏ |  | ❏ | ❏ |
| Kidney Problems | ❏ | ❏ |  | ❏ | ❏ |
| Myocardial Infarction | ❏ | ❏ |  | ❏ | ❏ |
| Congestive Heart Failure | ❏ | ❏ |  | ❏ | ❏ |
| Peripheral Vascular Disease | ❏ | ❏ |  | ❏ | ❏ |
| Cerebrovascular Disease | ❏ | ❏ |  | ❏ | ❏ |
| Dementia | ❏ | ❏ |  | ❏ | ❏ |
| Connective Tissue Disease | ❏ | ❏ |  | ❏ | ❏ |
| Leukemia | ❏ | ❏ |  | ❏ | ❏ |
| Malignant Lymphoma | ❏ | ❏ |  | ❏ | ❏ |
| Solid Tumor | ❏ | ❏ |  | ❏ | ❏ |
| Liver Disease | ❏ | ❏ |  | ❏ | ❏ |
| AIDS | ❏ | ❏ |  | ❏ | ❏ |
