# Supplementary material for: Sociodemographic, medical, health behavior, and psychosocial factors associated with COVID-19 diagnoses in the New Jersey cancer survivor cohort
Source: Cancer Causes Control. 2025 Apr 25;36(8):853–70. doi: 10.1007/s10552-025-01997-2 (PMC12289746; doi:10.1007/s10552-025-01997-2)
Supplement: Supplementary file 2 — Supplementary file2 (DOCX 1209 KB) [file 10552_2025_1997_MOESM2_ESM.docx]

## Supplementary material

**Supplementary** **Figure 1. Graphical representation of the unadjusted odds ratio for each variable that was studied. The OR and 95% CI are estimated from the univariate logistic regression. OR = Odds Ratio, CI = Confidence Interval, ICE = the Index of Concentration at the Extremes.**

**
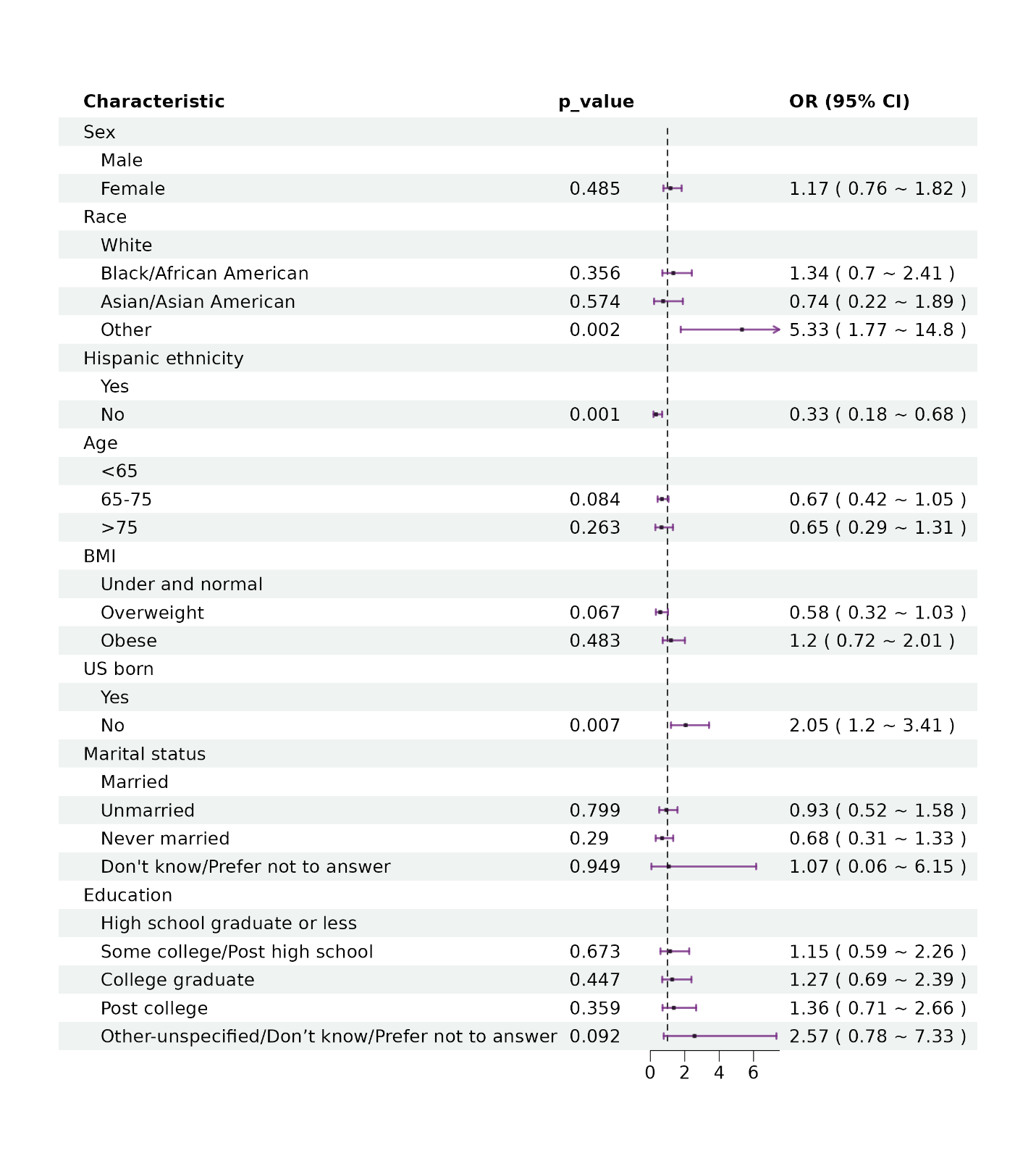
**

**
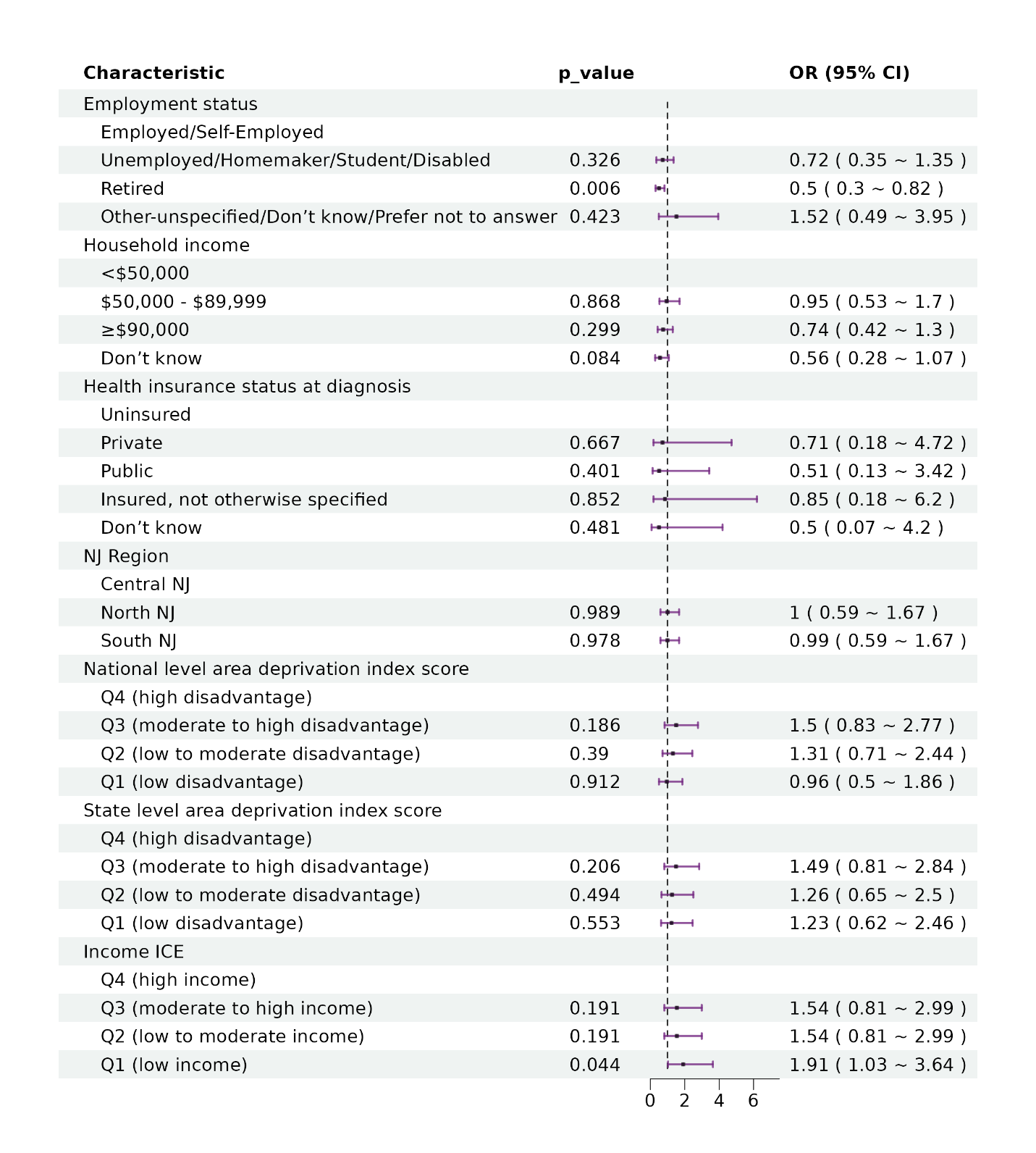

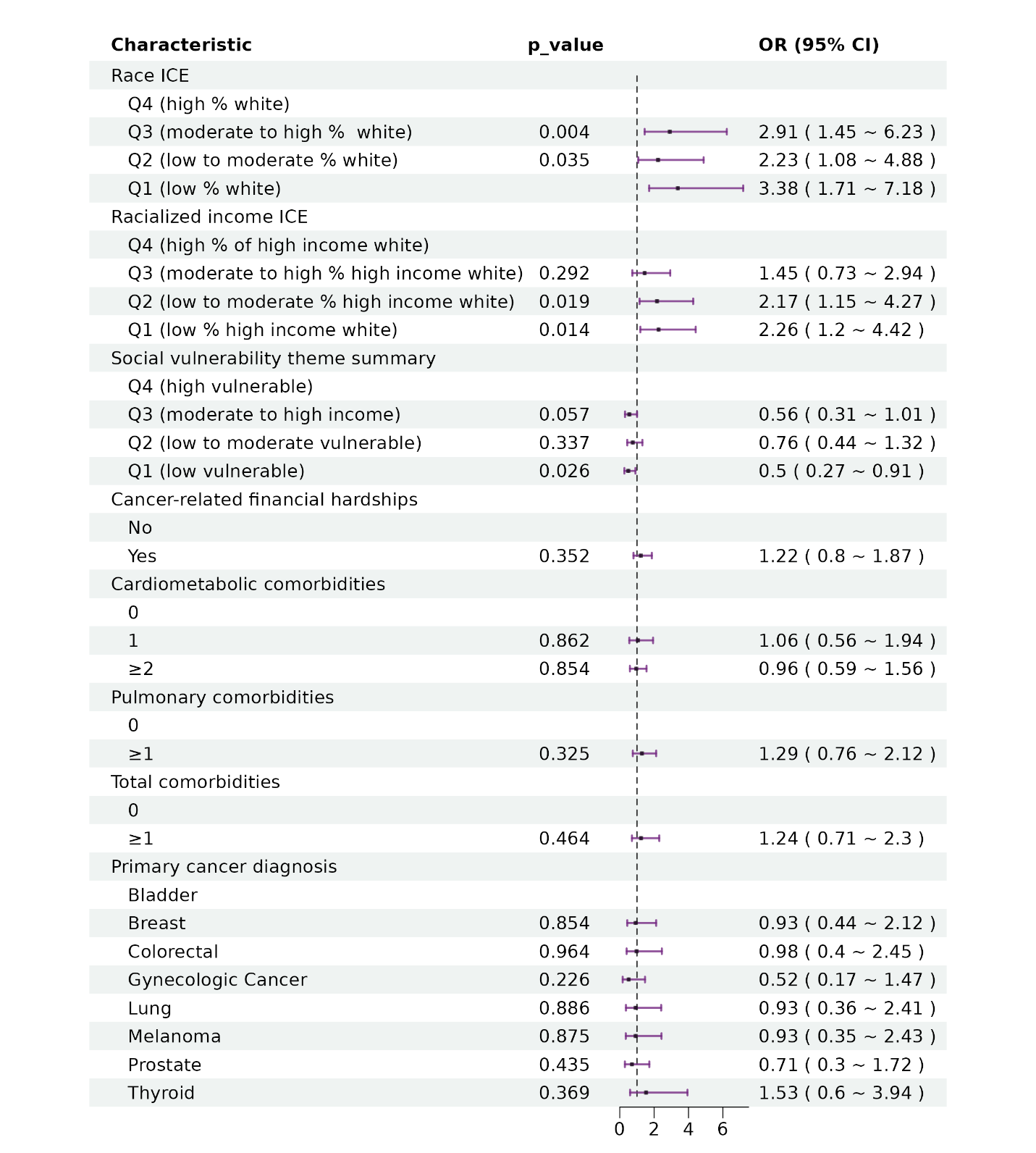

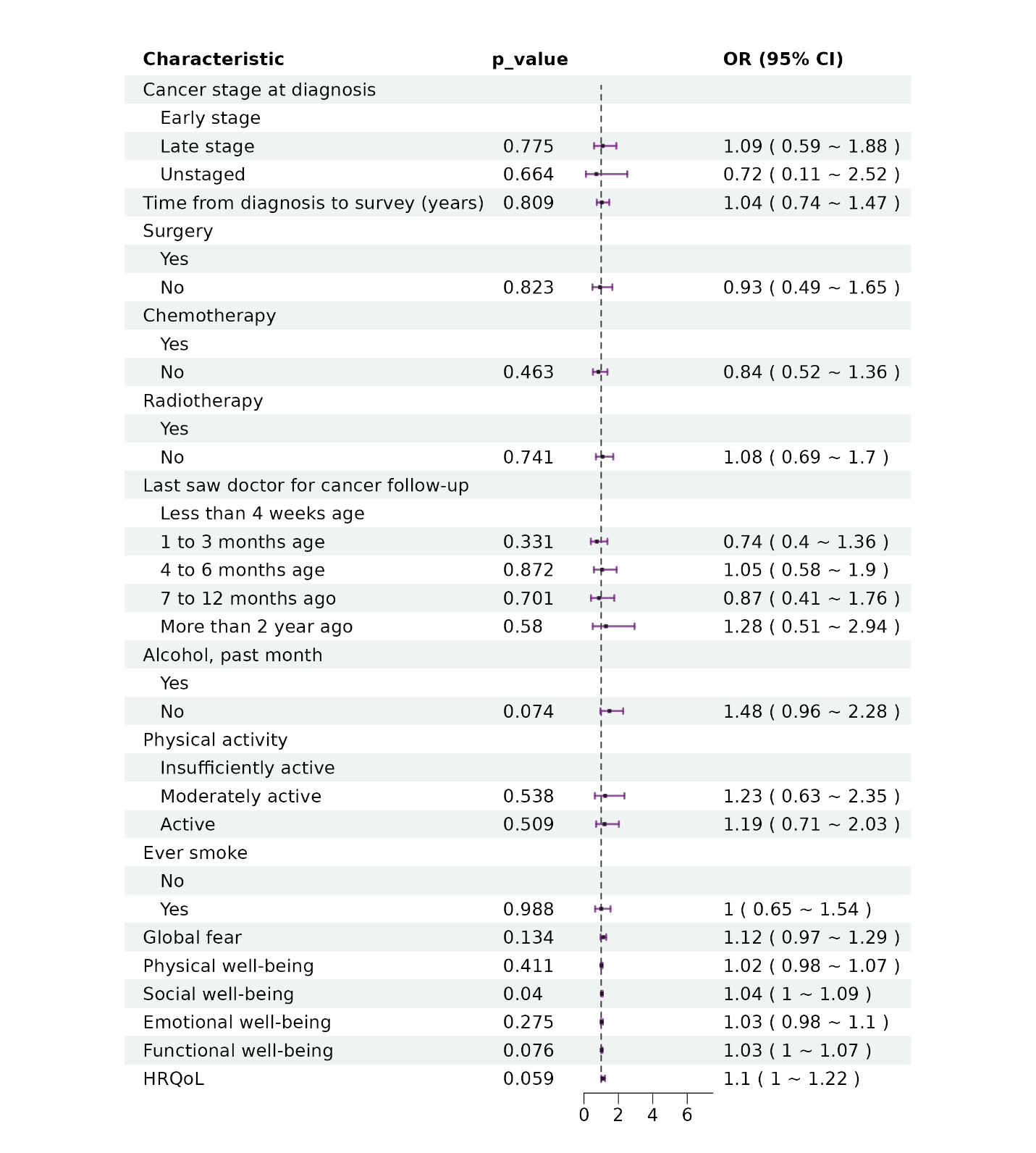
**


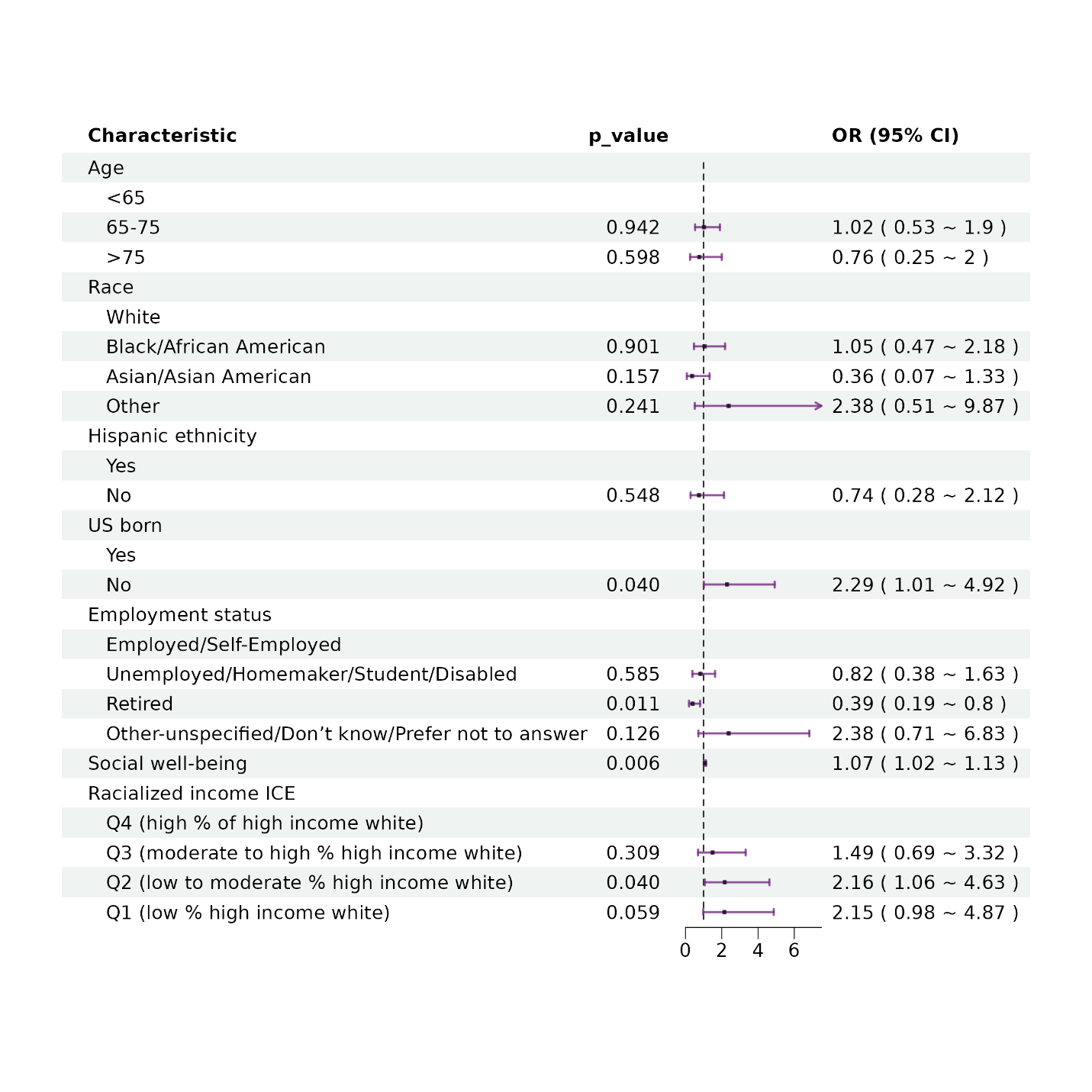


**Supplementary Table I. The results of multivariable logistic regression**

| Variable | OR^1^ | 95% CI^1^ | p-value |  |
| --- | --- | --- | --- | --- |
| Age |  |  |  |  |
| <65 | — | — |  |  |
| 65-75 | 1.02 | 0.53, 1.90 | >0.9 |  |
| >75 | 0.76 | 0.25, 2.00 | 0.6 |  |
| Race |  |  |  |  |
| White | — | — |  |  |
| Black/African American | 1.05 | 0.47, 2.18 | >0.9 |  |
| Asian/Asian American | 0.36 | 0.07, 1.33 | 0.2 |  |
| Other | 2.38 | 0.51, 9.87 | 0.2 |  |
| Hispanic ethnicity |  |  |  |  |
| Yes | — | — |  |  |
| No | 0.74 | 0.28, 2.12 | 0.5 |  |
| US born |  |  |  |  |
| Yes | — | — |  |  |
| No | 2.29 | 1.01, 4.92 | 0.04 |  |
| Employment status |  |  |  |  |
| Employed/Self-Employed | — | — |  |  |
| Unemployed/Homemaker/Student/Disabled | 0.82 | 0.38, 1.63 | 0.6 |  |
| Retired | 0.39 | 0.19, 0.80 | 0.011 |  |
| Other-unspecified/Don’t know/Prefer not to answer | 2.38 | 0.71, 6.83 | 0.13 |  |
| Social well-being | 1.07 | 1.02, 1.13 | 0.006 |  |
| Racialized income ICE |  |  |  |  |
| Q4 (high % of high income white vs low income black) | — | — |  |  |
| Q3 (moderate to high % high income white vs low income black) | 1.49 | 0.69, 3.32 | 0.3 |  |
| Q2 (low to moderate % high income white vs low income black) | 2.16 | 1.06, 4.63 | 0.040 |  |
| Q1 (low % high income white vs low income black) | 2.15 | 0.98, 4.87 | 0.059 |  |
| ^1^OR = Odds Ratio, CI = Confidence Interval | | | | |
